# Signal Detection and Machine Learning-Based Prediction of Cytokine Release Syndrome in B-Cell Maturation Antigen-Targeting Immunotherapies Using FAERS Data

**DOI:** 10.3390/ph19050669

**Published:** 2026-04-25

**Authors:** Suhyeon Moon, Dong-Won Kang, Yeo Jin Choi, Sooyoung Shin

**Affiliations:** 1 Department of Biohealth Regulatory Science, Graduate School, Ajou University, Suwon 16499, Republic of Korea; suhyeun28@ajou.ac.kr (S.M.); dwonkang@ajou.ac.kr (D.-W.K.); 2 Department of Pharmacy, College of Pharmacy, Ajou University, Suwon 16499, Republic of Korea; 3 College of Pharmacy and Institute of Integrated Pharmaceutical Science, Kyung Hee University, Seoul 02447, Republic of Korea

**Keywords:** cytokine release syndrome, CAR-T therapy, bispecific antibodies, pharmacovigilance, machine learning, FAERS

## Abstract

**Background/Objectives**: B-cell maturation antigen (BCMA)-directed immunotherapies, including chimeric antigen receptor T-cell (CAR-T) therapies and bispecific antibodies (BsAbs), have improved clinical outcomes in multiple myeloma. However, cytokine release syndrome (CRS) remains a major safety concern, and comparative real-world evidence across BCMA-directed agents remains limited. This study aimed to evaluate and compare CRS reporting patterns associated with BCMA-targeted CAR-T and BsAb therapies using the FDA Adverse Event Reporting System (FAERS) data and to identify predictors of CRS reporting using machine learning-based approaches. **Methods**: A pharmacovigilance analysis was conducted using FAERS reports from 2021 Q1 to 2025 Q3. Disproportionality analyses were performed using the reporting odds ratio (ROR), proportional reporting ratio (PRR), and information component (IC), and signals were considered present when predefined thresholds were met. Multivariable logistic regression was applied to estimate adjusted odds ratios (aORs) for CRS reporting while adjusting for demographic and reporting characteristics. Machine learning models, including XGBoost, LightGBM, and random forest were developed to predict CRS reporting. Model interpretability was assessed using SHapley Additive exPlanations (SHAP). **Results**: Among 4046 reports included in the final dataset, CAR-T therapies showed higher CRS reporting odds than BsAbs (aOR: 2.55, 95% CI: 2.16–3.01). Disproportionality analyses identified significant CRS signals for CAR-T therapies across all indices, whereas BsAbs did not meet signal detection thresholds. At the agent level, idecabtagene vicleucel was the only agent meeting all predefined signal detection criteria and exhibited the strongest reporting pattern in multivariable analysis (aOR: 6.96, 95% CI: 5.53–8.75). Among the evaluated models, LightGBM achieved the highest predictive test AUROC (0.762). SHAP analysis identified idecabtagene vicleucel, United States region, and reporting year as the most influential predictors of CRS reporting. **Conclusions**: CAR-T therapies, particularly idecabtagene vicleucel, exhibited higher CRS reporting odds than BsAbs, with substantial agent-level heterogeneity observed across BCMA-directed immunotherapies. Integrating pharmacovigilance and machine learning approaches may facilitate more individualized safety monitoring by identifying agent-specific differences in CRS risk among BCMA-targeted therapies.

## 1. Introduction

Multiple myeloma (MM) is a malignancy of plasma cells, characterized by the clonal proliferation of terminally differentiated B cells in the bone marrow, often resulting in end-organ damage such as anemia, renal impairment, hypercalcemia, and osteolytic bone disease [1,2]. It represents about 1–2% of all cancers and about 10% of hematologic malignancies worldwide [3,4]. According to recent epidemiological estimates, the global incidence of MM exceeds 187,000 new cases annually, with more than 121,000 MM-related deaths reported each year [5]. Despite therapeutic advances, MM remains incurable, with most patients eventually experiencing relapse and disease progression.

The introduction of novel agents has fundamentally reshaped the therapeutic landscape of MM over the past two decades. Standard frontline and relapsed/refractory regimens incorporate proteasome inhibitors (PIs) (bortezomib, carfilzomib, and ixazomib), immunomodulatory agents (thalidomide, lenalidomide, and pomalidomide), and monoclonal antibodies targeting CD38 (daratumumab and isatuximab) [6]. In transplant-eligible patients, these therapies are typically used in combination with high-dose chemotherapy followed by autologous stem cell transplantation (ASCT). Collectively, these strategies have significantly improved response rates and survival outcomes [7,8].

Nevertheless, the prognosis of MM remains suboptimal, particularly for patients harboring high-risk cytogenetic abnormalities or those with disease refractory to multiple classes of therapy. With each additional line of therapy, the depth and durability of response typically diminish, underscoring the critical importance of achieving deep, rapid, and sustainable responses in later lines of therapy [3,9]. In this context, minimal residual disease (MRD) negativity has been established as a key surrogate endpoint for long-term survival, emphasizing the need for more potent and targeted therapeutic approaches [10,11].

B-cell maturation antigen (BCMA), which belongs to the tumor necrosis factor receptor superfamily, is preferentially expressed on malignant plasma cells and represents a compelling therapeutic target in MM [12]. BCMA-directed immunotherapies, such as chimeric antigen receptor T-cell (CAR-T) therapies and bispecific antibodies (BsAbs), have demonstrated high response rates in heavily pretreated patients with relapsed or refractory MM. BCMA-targeting CAR-T therapies induce deep and durable responses through ex vivo engineered autologous T cells, while BsAbs redirect endogenous T cells to eliminate BCMA-expressing myeloma cells via dual antigen engagement [12,13,14].

Despite their remarkable clinical efficacy, BCMA-targeting immunotherapies are frequently associated with immune-mediated toxicities, most notably cytokine release syndrome (CRS) [15,16]. This involves a systemic inflammatory response associated with excessive immune activation, characterized by rapid T-cell proliferation and elevated circulating cytokines, such as interleukin (IL)-6, IL-10, tumor necrosis factor-α (TNF-α), and interferon-γ (IFN-γ) [17,18]. Clinical manifestations of CRS vary widely spanning a continuum from flu-like symptoms to severe complications, including hypotension, hypoxia, and multiorgan dysfunction. The incidence, severity, and temporal profile of CRS appear to differ between CAR-T and BsAb therapies, yet real-world comparative safety data remain limited [16].

Post-marketing pharmacovigilance databases provide a valuable opportunity to assess rare and severe adverse drug reactions in large, heterogeneous populations [19]. The U.S. Food and Drug Administration Adverse Event Reporting System (FAERS) is a spontaneous reporting system (SRS) widely used for signal detection and risk assessment of drug-associated toxicities [20]. To date, no study has evaluated the comparative risk of CRS between BCMA-targeted CAR-T and BsAb immunotherapies using FAERS data with an integrated pharmacovigilance and machine learning approach.

This study aimed to conduct a comprehensive pharmacovigilance analysis to compare CRS reporting associated with BCMA-targeted CAR-T and BsAb therapies and to develop a machine learning-based model to identify factors associated with CRS in real-world clinical practice. By integrating multivariable regression with predictive modeling, we sought to generate clinically relevant evidence to support improved risk stratification and management of CRS in patients with MM.

## 2. Results

### 2.1. Case Selection and Data Processing

**Figure 1:**
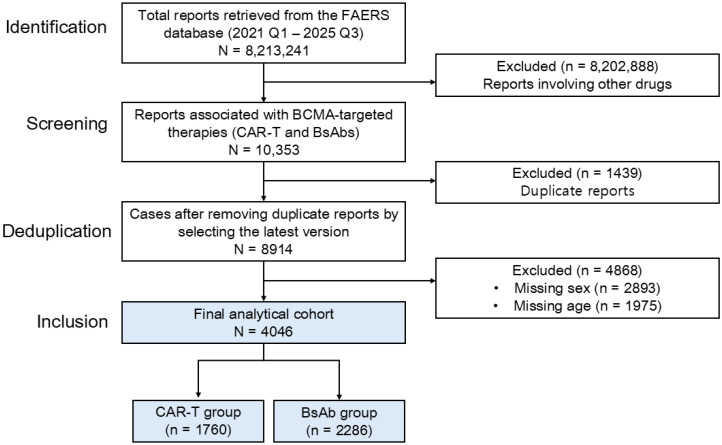
Flowchart of the adverse event report selection process for CAR-T cell therapy- and BsAb-associated reports. Abbreviations: FAERS, FDA Adverse Event Reporting System; BCMA, B-cell maturation antigen; CAR-T, chimeric antigen receptor T-cell; BsAb, bispecific antibody.

A total of 8,213,241 adverse event (AE) reports retrieved from the FAERS database from 2021 Q1 to 2025 Q3 were initially identified. From this initial dataset, 10,353 reports associated with BCMA-targeted therapies were retrieved for screening. To ensure data integrity, 1439 duplicate reports were removed by retaining the most recent version of each case. Among the remaining 8914 reports, 4868 were further excluded due to missing age, sex, or reporter region. Consequently, a final analytical dataset of 4046 reports was established (Figure 1). Linvoseltamab was not included as a study drug because only one FAERS report was identified during the study period.

### 2.2. Demographic Characteristics of the Study Dataset

The final analytical dataset comprised 4046 AE reports, including 1760 (43.5%) associated with CAR-T cell therapies and 2286 (56.5%) associated with BsAbs (Table 1). Both treatment groups were predominantly composed of older adults aged ≥65 years, with a higher proportion in the BsAb group (63.2%) than in the CAR-T group (58.2%). Significant geographical and reporter-type disparities were observed between the two modalities. Most CAR-T reports originated from the United States (79.2%), whereas BsAb reports showed a more global distribution, with 72.9% originating from non-United States regions. Reporting patterns also differed significantly; consumers submitted 52.6% of CAR-T reports, while healthcare professionals filed 72.9% of BsAb reports. Furthermore, the prevalence of polypharmacy (≥5 medications) was more than twice as high in the BsAb group compared with the CAR-T group (43.2% vs. 19.9%). All baseline characteristics differed significantly between groups (*p* < 0.001).

### 2.3. Disproportionality Analysis for CRS

**Figure 2:**
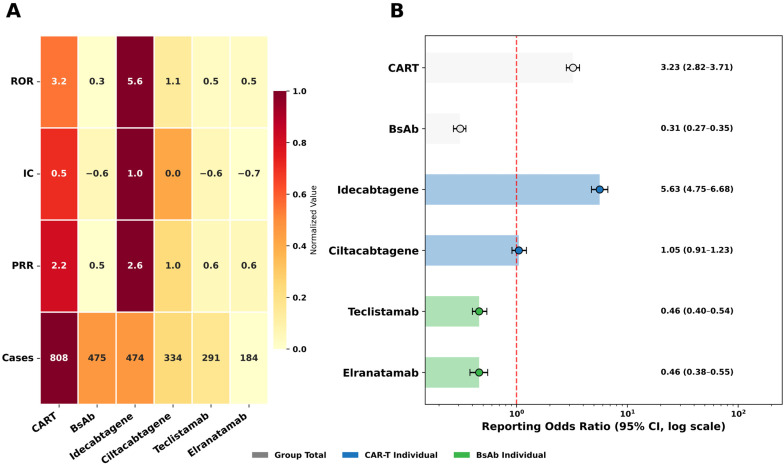
Disproportionality analysis of CRS for BCMA-targeted immunotherapies. (**A**) Heatmap of ROR, IC, and PRR across treatment groups and individual agents. (**B**) Forest plot of RORs with 95% confidence intervals from internal comparisons within the BCMA-targeted groups. The vertical line indicates ROR = 1. Abbreviations: ROR, reporting odds ratio; IC, information component; PRR, proportional reporting ratio; CAR-T, chimeric antigen receptor T-cell; BsAb, bispecific antibody; CI, confidence interval; CRS, cytokine release syndrome; BCMA, B-cell maturation antigen.

The disproportionality analysis revealed distinct reporting patterns for CRS between the two treatment modalities (Figure 2A). At the group level, CAR-T therapies demonstrated a significant disproportional reporting signal across all indices (reporting odds ratio [ROR]: 3.2, information component [IC]: 0.5, proportional reporting ratio [PRR]: 2.2), whereas the BsAb group did not meet the predefined thresholds for signal detection. At the individual agent level, idecabtagene vicleucel was the only agent exhibiting a significant signal (ROR: 5.6, IC: 1.0, PRR: 2.6), while ciltacabtagene autoleucel, teclistamab, and elranatamab did not satisfy the criteria for signal detection (Figure 2B).

### 2.4. Multivariable Logistic Regression Analysis of CRS Reporting

A multivariable logistic regression analysis was performed to assess factors associated with CRS reporting. Among reports associated with CAR-T therapies, CRS was reported in 808 of 1760 cases (45.9%), nearly twice the proportion observed in BsAb therapies (475 of 2286, 20.8%) (Table 2). After adjustment for age group, sex, polypharmacy status, geographic region, reporter type, report year, and MM indication, CAR-T therapies were associated with significantly higher odds of CRS reporting compared with BsAb therapies (adjusted odds ratio [aOR]: 2.55, confidence interval [CI]: 2.16–3.01).

At the individual agent level, idecabtagene vicleucel, despite having fewer reports than ciltacabtagene autoleucel (734 vs. 1026), showed the highest CRS reporting rate among all agents (64.6%) and the highest adjusted reporting odds (aOR: 6.96, 95% CI: 5.53–8.75), followed by ciltacabtagene autoleucel (aOR: 1.43, 95% CI: 1.17–1.75), compared with the BsAb reference group (Table 2). Among the BsAbs, teclistamab was associated with higher reporting odds (aOR: 1.28, 95% CI: 1.02–1.60), compared with elranatamab.

Stratified analyses showed that higher reporting odds for CAR-T therapies compared with BsAbs were consistently observed across all subgroups (Supplementary Table S1), including geographic region, age, sex, reporter type, and polypharmacy status (all *p* < 0.001). Notably, consumer reporters showed substantially higher odds than those submitted by healthcare professionals, with aORs of 4.37 and 1.55, respectively. Higher reporting odds were also observed irrespective of polypharmacy status, although the magnitude was lower in the polypharmacy group than in the non-polypharmacy group (aORs of 2.02 and 2.95, respectively).

### 2.5. Predictive Performance of Machine Learning Models

Table 3 summarizes the predictive performance of the evaluated machine learning models. Light gradient boosting machine (LightGBM) achieved the highest test area under the receiver operating characteristic curve (AUROC) of 0.762, along with the highest area under the precision-recall curve (PR-AUC) and harmonic mean of precision and recall (F1-score) of 0.621 and 0.601, respectively (test-set precision = 0.496). Random forest (RF) showed a comparable test AUROC of 0.753 with the highest recall among the tree-based models (0.805). Extreme gradient boosting (XGBoost) also showed comparable discriminative performance, with a test AUROC of 0.750. Logistic regression (LR), despite achieving the highest overall recall of 0.829, showed the lowest discriminative performance, with a test AUROC of 0.726. Receiver operating characteristic (ROC) and precision–recall (PR) curves for the four models are shown in Supplementary Figure S1. To evaluate the consistency of model performance across different data splits, we implemented 100 independent train–test splits using distinct random seeds. LightGBM yielded a mean test AUROC of 0.737 ± 0.017 and a PR-AUC of 0.590 ± 0.024, while the remaining models demonstrated comparable stability (Supplementary Table S6). The cross-validation AUROCs (Table 3; range 0.703–0.732) were comparable to the test-set AUROCs (0.726–0.762).

### 2.6. Sensitivity Analyses

Using a least absolute shrinkage and selection operator (LASSO)-reduced set of eight features (Supplementary Table S2), XGBoost and LightGBM both achieved an AUROC of 0.758, followed by RF and LR with AUROCs of 0.749 and 0.724, respectively (Supplementary Figure S2). To assess the robustness of the imbalance-handling approach, class re-weighting was compared with the Synthetic Minority Oversampling Technique (SMOTE) and random undersampling using the primary split (Supplementary Table S7). Among the three strategies, class re-weighting showed the best overall discrimination performance, with LightGBM achieving the highest AUROC (0.762) and PR-AUC (0.621). Performance differences between strategies were small, with maximum absolute differences of 0.015 in AUROC and 0.018 in PR-AUC. Model rankings were also consistent across strategies, indicating that the main conclusions were stable regardless of the imbalance-handling method used.

### 2.7. Feature Importance and Directionality (SHAP Analysis)

**Figure 3:**
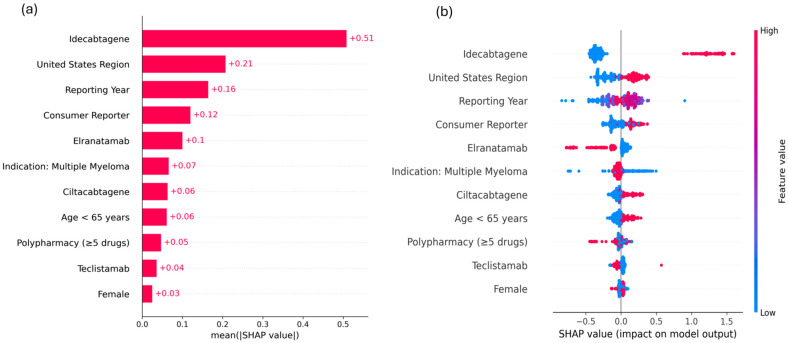
SHAP analysis of feature contributions in the LightGBM model for CRS prediction. (**a**) SHAP importance plot showing mean absolute SHAP values for each feature. (**b**) SHAP beeswarm plot showing the distribution of SHAP values across individual reports. Abbreviations: SHAP, SHapley Additive exPlanations; CRS, cytokine release syndrome; LightGBM, light gradient boosting machine.

Global feature importance was assessed using mean absolute SHapley Additive exPlanations (SHAP) values to identify key drivers of the model predictions (Figure 3a). Idecabtagene showed the highest importance (mean |SHAP| = 0.51), followed by United States region (0.21), reporting year (0.16), and consumer reporter (0.12). Elranatamab, MM indication, ciltacabtagene, and age <65 years showed relatively modest contributions.

The directional effects of SHAP values are illustrated in the summary plot (Figure 3b). Idecabtagene and United States region were predominantly associated with positive SHAP values, indicating an increased likelihood of CRS reporting. In contrast, elranatamab showed predominantly negative SHAP values, suggesting a lower likelihood of CRS reporting.

## 3. Discussion

In this FAERS-based comparative analysis of CRS reporting among BCMA-targeted immunotherapies for MM, CAR-T therapies met the predefined signal detection thresholds across all disproportionality indices, whereas BsAbs did not. Consistently, multivariable logistic regression showed that CAR-T therapies were associated with higher reporting odds of CRS than BsAbs after adjustment for demographic and reporting-related factors (aOR: 2.55, 95% CI: 2.16–3.01). At the individual agent level, idecabtagene vicleucel showed the highest adjusted reporting odds of CRS (aOR: 6.96, 95% CI: 5.53–8.75). In the SHAP analysis, it also had the highest mean absolute SHAP value (0.51) and was predominantly associated with positive SHAP values in the summary plot.

Our findings are consistent with prior clinical and pharmacovigilance studies; however, the present analysis provides a more focused evaluation of CRS reporting across currently approved BCMA-targeted therapies in MM. CRS has been recognized as a frequent and clinically important toxicity across both CAR-T therapies and BsAbs, in pivotal trials including idecabtagene vicleucel in KarMMa and teclistamab in MajesTEC-1 [10,14]. A prior meta-analysis of clinical trial data also suggested a greater CRS burden with CAR-T therapies than with BsAbs in BCMA-directed settings [16,21]. In the present analysis, a similar pattern was observed in real-world reporting data, with CRS reported in 45.9% of CAR-T-associated cases and 20.8% of BsAb-associated cases, along with higher adjusted reporting odds for CAR-T therapies compared with BsAbs. At the individual agent level, idecabtagene vicleucel showed the strongest CRS reporting odds among the evaluated therapies, consistent with its higher ROR reported in prior FAERS-based analyses of BCMA-directed immunotherapies [22,23]. The present study further extends previous findings by focusing specifically on CRS and evaluating whether these reporting patterns were maintained after covariate adjustment and machine learning-based analyses.

Agent-level differences should be considered when interpreting these findings. Within the CAR-T class, idecabtagene vicleucel showed higher adjusted reporting odds of CRS than ciltacabtagene autoleucel (aOR: 6.96 vs. 1.43; both *p* < 0.001) and was the only agent that met the predefined disproportionality thresholds at the individual level. Differences in CRS reporting were also observed between the evaluated BsAbs, with teclistamab showing higher adjusted reporting odds than elranatamab (aOR: 1.28; *p* = 0.037). These patterns are consistent with clinical trial data demonstrating that the frequency and severity of CRS vary across individual BCMA-directed therapies [10,14]. Taken together, these findings highlight substantial heterogeneity in CRS reporting across individual BCMA-directed therapies.

Geographic and reporter-related factors should also be considered when interpreting these findings. In stratified analyses, higher CRS reporting odds for CAR-T therapies relative to BsAbs were consistently observed across both United States and non-United States reports. Differences by reporter type were particularly pronounced, with aORs of 4.37 for consumer reports and 1.55 for healthcare professional reports (both *p* < 0.001). Higher reporting odds were also observed regardless of polypharmacy status, with aORs of 2.95 in reports without polypharmacy and 2.02 in those with polypharmacy (both *p* < 0.001).

Machine learning analyses were conducted to assess whether the findings from the disproportionality and regression analyses were consistent across a different analytic framework. Among the evaluated models, LightGBM achieved the highest test AUROC (0.762). SHAP results were aligned with the main findings, with idecabtagene vicleucel contributing prominently to model predictions. Sensitivity analysis using LASSO-based feature selection showed only modest changes in predictive performance, with an AUROC of 0.758 for both XGBoost and LightGBM. Overall, the consistent findings across signal detection, multivariable regression, and machine learning analyses support continued safety surveillance of BCMA-directed immunotherapies in real-world settings. However, prospective studies incorporating more detailed patient-level and treatment-related information are needed to further evaluate these observations and to better characterize potential agent-specific differences in CRS reporting.

### Limitations

Our study has several limitations that should be considered when interpreting these study findings. First, as this analysis is based on the FAERS database, it is subject to inherent biases such as under-reporting and stimulated reporting following the approval of newly introduced therapies [19,24]. Second, the reliance on spontaneous reports means that the observed associations cannot establish a definitive causal relationship [25]. Third, although the relatively high proportion of consumer reports in our dataset may reflect patient-reported experiences in real-world settings, it may also introduce variability compared with reports submitted by healthcare professionals. Fourth, the absence of a denominator for exposed patients prevents estimation of the true incidence of CRS. In addition, 4868 reports with missing age, sex, or reporter region were excluded, which may have introduced selection bias and affected generalizability. Finally, detailed clinical information such as CRS severity or grading, was often unavailable in this dataset, limiting stratified analyses according to CRS severity.

## 4. Materials and Methods

### 4.1. Data Source and Study Population

A retrospective analysis was conducted using data from the FAERS database between the first quarter of 2021 and the third quarter of 2025. This period was set to cover post-approval reporting for the four BCMA-directed immunotherapies included in this study, following the FDA approval dates for idecabtagene vicleucel (26 March 2021) [26], ciltacabtagene autoleucel (28 February 2022) [27], teclistamab-cqyv (25 October 2022) [28], and elranatamab-bcmm (14 August 2023) [29]. The study was granted an exemption by the Institutional Review Board of Ajou University on 26 December 2025 (IRB No. 202512-HB-EX-001). 

### 4.2. Inclusion and Exclusion Criteria

Data processing followed the FDA recommendations for the use of FAERS data. Reports with invalid demographic information, such as missing sex or age ≤ 0, were excluded. To eliminate duplicate reports, only the latest version of each case was kept in accordance with the FDA’s recommended deduplication procedure [30,31,32]. Study drugs were identified from the DRUG table (drug information) and restricted to those coded as primary or secondary suspects [19]. Clinical variables were extracted from the INDI (indication), OUTC (patient outcomes), and THER (therapy start and end dates) tables to construct the final analytical dataset.

### 4.3. Exposure and Outcome Definitions

Reports were categorized into two groups based on the suspected drug exposure: BCMA-directed CAR-T cell therapies (idecabtagene vicleucel and ciltacabtagene autoleucel) and BsAbs (teclistamab and elranatamab) [33,34]. The primary outcome was CRS reporting, identified using MedDRA (version 28.0) Preferred Terms “cytokine release syndrome” and “cytokine storm”. Concomitant medications were counted per report, and polypharmacy was defined as ≥5 drugs. Other covariates included MM indication, age, sex, reporter type, and geographic region [35].

### 4.4. Disproportionality Analysis (Signal Detection)

To detect potential reporting signals of CRS associated with BCMA-targeted therapies, disproportionality analyses were performed using the ROR, PRR, and IC [36,37]. These measures compare the reporting frequency of CRS for a specific drug with that for all other drugs in the database. Signals were defined as ROR with 95% CI lower bound > 1, PRR ≥ 2 accompanied by χ^2^ ≥ 4, or IC with 95% CI lower bound > 0 [31,38]. Detailed calculation formulas and the criteria for signal detection are summarized in Supplementary Table S3.

### 4.5. Multivariable Logistic Regression

The aORs for CRS reporting were estimated by multivariable logistic regression. The models were adjusted for age, sex, geographic region, reporter type, report year, and MM indication [39]. Three analytical approaches were applied: (1) group-level comparison between CAR-T and BsAb therapies; (2) CAR-T agent-level comparisons with the BsAb reference group; and (3) comparison between individual BsAbs. In addition, stratified analyses were performed across predefined subgroups, including region, age, sex, reporter type, and polypharmacy status, defined as non-polypharmacy (<5) and polypharmacy (≥5 concomitant medications). Statistical significance was defined as a 95% CI that did not include 1.0 [35].

### 4.6. Machine Learning-Based Predictive Model Development

Predictive modeling was performed using an 80:20 train-test split to predict CRS reporting. Four machine learning-based classification algorithms were implemented: RF [40], LR, XGBoost [41] and LGBM [42]. Each model’s hyperparameters were tuned by grid search with 5-fold cross-validation on the training data [43,44], and the evaluated hyperparameter ranges and selected values are presented in Supplementary Table S4. The models incorporated 11 clinical features such as drug type, demographic variables, and polypharmacy level, with the complete feature list provided in Supplementary Table S5. To address class imbalance, class re-weighting was applied to the minority class, corresponding to CRS-positive reports. Discrimination was assessed by AUROC and F1-score, and the classification threshold was chosen from the precision-recall curve [45,46]. To assess the stability of model performance across different data splits, the 80:20 stratified split was repeated 100 times using random seeds of 0–99, and the mean ± SD of each test-set metric was recorded. All statistical analyses were performed using Python (version 3.12.12).

### 4.7. Sensitivity Analysis

First, LASSO regression was additionally applied to the training set to derive a reduced feature set [47,48]. The four machine-learning models were re-evaluated using this subset as a supplementary sensitivity analysis to assess AUROC-based discrimination after feature reduction [49].

Second, the class re-weighting strategy was compared with SMOTE [50] and random undersampling of the majority class [51]. Both alternatives were applied only to the training set of the primary split, leaving the held-out test set unchanged, and the four models were refit with the same hyperparameters selected from the original 5-fold cross-validation.

### 4.8. Framework for Model Interpretability

To enhance the interpretability of the predictive models, SHAP values were used to quantify and rank the contribution of each clinical feature to CRS prediction [52,53]. This approach enabled the identification of key predictors and their relative contributions to the predicted probability of CRS across the different models.

## 5. Conclusions

CAR-T therapies showed higher CRS reporting odds than BsAbs, with idecabtagene vicleucel exhibiting the strongest odds among the evaluated agents. Substantial agent-level differences in CRS reporting were observed across BCMA-directed immunotherapies, indicating substantial heterogeneity in safety profiles even within the same therapeutic class. By incorporating multivariable regression and machine learning-based approaches, this study demonstrated that CRS reporting is influenced by both agent-specific and reporting-related factors, with consistent findings across all evaluated models. These findings highlight the importance of considering agent-specific safety profiles when monitoring CRS risk in patients receiving BCMA-directed immunotherapies.

## Figures and Tables

**Table 1:** Baseline characteristics of adverse event reports for BCMA-targeted therapies.

Characteristic	CAR-T Reports (n = 1760)	BsAb Reports (n = 2286)	*p*-Value
Report year, n (%)			<0.001
≤2021	94 (5.3)	5 (0.2)	
2022	337 (19.1)	144 (6.3)	
2023	381 (21.6)	762 (33.3)	
2024	512 (29.1)	677 (29.6)	
2025	436 (24.8)	698 (30.5)	
Region, n (%)			<0.001
United States	1394 (79.2)	620 (27.1)	
Non-United States	366 (20.8)	1666 (72.9)	
Age group, n (%)			<0.001
<65 years	735 (41.8)	842 (36.8)	
≥65 years	1025 (58.2)	1444 (63.2)	
Sex, n (%)			<0.001
Female	692 (39.3)	1042 (45.6)	
Male	1068 (60.7)	1244 (54.4)	
Reporter type, n (%)			<0.001
Healthcare professional *	834 (47.4)	1666 (72.9)	
Consumer	926 (52.6)	620 (27.1)	
Indication, n (%)			<0.001
Multiple myeloma	1470 (83.5)	1966 (86.0)	
Others	290 (16.5)	320 (14.0)	
Polypharmacy status, n (%)			<0.001
<5 medications	1410 (80.1)	1298 (56.8)	
≥5 medications	350 (19.9)	988 (43.2)	

* Consisted of physician, pharmacist, and other healthcare professionals. Abbreviations: CAR-T, chimeric antigen receptor T-cell therapy; BsAb, bispecific antibody.

**Table 2:** Multivariable logistic regression analysis of CRS reporting at the group and individual drug levels.

Drug	Overall Reports, n	CRS Reports, n (%)	Reference	Overall Reports, n	CRS Reports, n (%)	Crude OR (95% CI)	Adjusted OR (95% CI) ^1^	*p*-Value ^2^
CAR-T cell therapies	1760	808 (45.9)	BsAbs	2286	475 (20.8)	3.24 (2.82–3.71)	2.55 (2.16–3.01)	<0.001
Idecabtagene vicleucel	734	474 (64.6)	BsAbs	2286	475 (20.8)	6.95 (5.79–8.34)	6.96 (5.53–8.75)	<0.001
Ciltacabtagene autoleucel	1026	334 (32.6)	BsAbs	2286	475 (20.8)	1.84 (1.56–2.17)	1.43 (1.17–1.75)	<0.001
Teclistamab	1361	291 (21.4)	Elranatamab	924	184 (19.9)	1.04 (0.88–1.22)	1.28 (1.02–1.60)	0.037

^1^ Adjusted for age, sex, region, reporter type, polypharmacy, report year, and MM indication. ^2^ *p*-values for adjusted comparisons. Abbreviations: CRS, cytokine release syndrome; OR, odds ratio; CI, confidence interval; CAR-T, chimeric antigen receptor T-cell therapy; BsAbs, bispecific antibodies.

**Table 3:** Predictive performance comparison of machine learning models.

Model	CV AUROC (Mean ± SD)	Test AUROC	PR-AUC	F1-Score	Recall	Precision
LightGBM	0.729 ± 0.011	0.762	0.621	0.601	0.763	0.496
Random Forest	0.732 ± 0.011	0.753	0.603	0.589	0.805	0.464
XGBoost	0.729 ± 0.013	0.750	0.606	0.588	0.759	0.480
Logistic Regression	0.703 ± 0.013	0.726	0.565	0.565	0.829	0.429

Abbreviations: CV, cross-validation; AUROC, area under the receiver operating characteristic curve; SD, standard deviation; PR-AUC, area under the precision–recall curve; F1-score, harmonic mean of precision and recall; LightGBM, light gradient boosting machine; XGBoost, extreme gradient boosting.

## Data Availability

The original data presented in the study are openly available in the FDA Adverse Event Reporting System (FAERS) at https://fis.fda.gov/extensions/FPD-QDE-FAERS/FPD-QDE-FAERS.html, accessed on 17 December 2025.

## Supplementary Materials

The following supporting information can be downloaded at: https://www.mdpi.com/article/10.3390/ph19050669/s1, Table S1: Stratified multivariable analysis of CRS reporting; Table S2: List of features selected via LASSO regularization; Table S3: Definitions and signal detection criteria for disproportionality analyses; Table S4: Hyperparameter search space and selected best values; Table S5: Full list of clinical features used in modeling; Table S6: Test-set performance metrics across 100 repeated train-test splits; Table S7: Comparison of class imbalance handling strategies across four machine learning models; Figure S1: ROC and PR curves of machine learning models for CRS prediction; Figure S2: ROC curves of machine learning models for predicting CRS using LASSO-selected features.
