# Supplementary material for: Signal Detection and Machine Learning-Based Prediction of Cytokine Release Syndrome in B-Cell Maturation Antigen-Targeting Immunotherapies Using FAERS Data
Source: Pharmaceuticals (Basel). 2026 Apr 25;19(5):669. doi: 10.3390/ph19050669 (PMC13209349; doi:10.3390/ph19050669)
Supplement: Supplementary file 1 [file pharmaceuticals-19-00669-s001.zip › pharmaceuticals-4263685-supplementary.pdf]

Supplementary Materials

Table S1. Stratified multivariable analysis of CRS reporting: CAR-T vs. BsAb

Category	Subgroup	CAR-T		BsAb		Crude OR (95% CI)	Adjusted OR[1] (95% CI)	p-value[2]
		Reports, n	CRS, n (%)	Reports, n	CRS, n (%)			
Overall		1,760	808 (45.9)	2,286	475 (20.8)	3.24 (2.82–3.71)	2.55 (2.16–3.01)	<0.001
Region	United States	1,394	672 (48.2)	620	157 (25.3)	2.74 (2.23–3.38)	2.52 (2.02–3.16)	<0.001
	Non-US	366	136 (37.2)	1,666	318 (19.1)	2.51 (1.96–3.20)	3.06 (2.33–4.01)	<0.001
Age	<65 years	735	328 (44.6)	842	196 (23.3)	2.66 (2.14–3.30)	2.43 (1.88–3.15)	<0.001
	≥65 years	1,025	480 (46.8)	1,444	279 (19.3)	3.68 (3.07–4.40)	2.61 (2.09–3.26)	<0.001
Sex	Male	1,068	473 (44.3)	1,244	250 (20.1)	3.16 (2.63–3.80)	2.48 (1.99–3.10)	<0.001
	Female	692	335 (48.4)	1,042	225 (21.6)	3.41 (2.76–4.20)	2.67 (2.07–3.45)	<0.001
Reporter Type	Healthcare Professional[3]	834	281 (33.7)	1,666	336 (20.2)	2.01 (1.67–2.42)	1.55 (1.24–1.95)	<0.001
	Consumer	926	527 (56.9)	620	139 (22.4)	4.57 (3.63–5.75)	4.37 (3.32–5.75)	<0.001
Polypharmacy	<5 medications	1,410	697 (49.4)	1,298	278 (21.4)	3.59 (3.03–4.25)	2.95 (2.39-3.63)	<0.001
	≥5 medications	350	111 (31.7)	988	197 (19.9)	1.86 (1.42-2.45)	2.02 (1.49-2.75)	<0.001

[1]Each stratified analysis adjusted for all covariates except the stratification variable itself.

[2]*p*-values for adjusted comparisons.

[3]Included physicians, pharmacists and other healthcare professionals.

Abbreviations: CRS, cytokine release syndrome; CAR-T, chimeric antigen receptor T-cell therapy; BsAb, bispecific antibody; OR, odds ratio; CI, confidence interval.

Table S2. List of features selected via LASSO regularization

Category	Variable	Description
Temporal	Report_Year	Year of adverse event report submission
Clinical	Indication_MM	Multiple myeloma as reported indication
	Polypharmacy_ge_5	Concurrent use of ≥5 medications
Drug-specific	Specific_Drug_Idecabtagene	Use of idecabtagene vicleucel
	Specific_Drug_Teclistamab	Use of teclistamab
	Specific_Drug_Elranatamab	Use of elranatamab
Reporter	Reporter_Type_Consumer	Report submitted by non-healthcare professionals*
Geographic	Region_US	Reports originating from the United States

*Defined as reporters not classified as healthcare professionals, including patients and other non-medical reporters.

Abbreviations: LASSO, least absolute shrinkage and selection operator; MM, multiple myeloma; US, United States.

Table S3. Definitions and signal detection criteria for disproportionality analyses

Index	Equation / Calculation	Signal Detection Criteria
ROR	$(a/c) / (b/d) = (a \times d) / (b \times c)$	Lower limit of 95% CI > 1
PRR	$[a / (a + b)] / [c / (c + d)]$	PRR ≥ 2 and $\chi^2 \geq 4$
IC	$\log2 \{ a \times (a + b + c + d) / [(a + b) \times (a + c)] \}$	Lower limit of 95% CI > 0

Notes: a = reports with suspect drug + target AE; b = suspect drug + other AEs; c = other drugs + target AE; d = other drugs + other AEs.

Abbreviations: ROR, reporting odds ratio; PRR, proportional reporting ratio; IC, information component; CI, confidence interval; AE, adverse event.

Table S4. Hyperparameter search space and selected best values for grid search with 5-fold cross-validation

Model	Hyperparameter	Search space	Selected value
LR	penalty	l1	l1
LR	solver	liblinear	liblinear
LR	class_weight	balanced	balanced
LR	C	0.001, 0.01, 0.05, 0.1, 0.5, 1.0, 5.0, 10.0	5.0
LR	max_iter	5000	5000
RF	n_estimators	100, 300, 500, 1000	1000
RF	max_depth	None, 5, 10, 20	5
RF	min_samples_split	2, 5, 10	2
RF	min_samples_leaf	2, 4, 10	2
RF	class_weight	balanced	balanced
XGBoost	n_estimators	100, 300, 500	100
XGBoost	learning_rate	0.01, 0.05, 0.1	0.1
XGBoost	max_depth	3, 5, 7	3
XGBoost	scale_pos_weight	ratio of negative to positive samples in the training set	ratio of negative to positive samples in the training set
LightGBM	n_estimators	100, 300, 500	300
LightGBM	learning_rate	0.01, 0.05, 0.1	0.01
LightGBM	max_depth	3, 5, 7	5
LightGBM	scale_pos_weight	ratio of negative to positive samples in the training set	ratio of negative to positive samples in the training set

Abbreviations: LR, logistic regression; RF, random forest; XGBoost, extreme gradient boosting; LGBM, light gradient boosting machine.

Table S5. Full list of clinical features used in modeling

Category	Feature Name	Description
Time	Report_Year	Year of adverse event reporting
Indication	Indication_MM	Indication: Multiple Myeloma
Drug	Specific_Drug_Ciltacabtagene	BCMA-directed CAR-T (Cilta-cel)
Drug	Specific_Drug_Elranatamab	BCMA-directed BsAb (Elranatamab)
Drug	Specific_Drug_Idecabtagene	BCMA-directed CAR-T (Ide-cel)
Drug	Specific_Drug_Teclistamab	BCMA-directed BsAb (Teclistamab)
Demographics	Sex_cleaned_F	Gender: Female
Demographics	Age_under_65	Age: Less than 65 years
Reporter	Reporter_Type_Consumer	Reporter: Consumer or non-HCP
Region	Region_US	Region: Within the United States
Polypharmacy	Polypharmacy_ge_5	Concomitant medications: 5 or more

Abbreviations: MM, multiple myeloma; BCMA, B-cell maturation antigen; CAR-T, chimeric antigen receptor T cell; BsAb, bispecific antibody; HCP, healthcare professional; US, United States.

Table S6. Test-set performance across 100 repeated 80:20 train-test splits (mean ± standard deviation).

Model	ROC-AUC	PR-AUC	F1-score	Recall	Precision
Logistic Regression	0.712 ± 0.019	0.554 ± 0.025	0.556 ± 0.019	0.661 ± 0.103	0.496 ± 0.065
Random Forest	0.741 ± 0.018	0.590 ± 0.024	0.579 ± 0.018	0.690 ± 0.087	0.512 ± 0.064
XGBoost	0.735 ± 0.017	0.590 ± 0.024	0.576 ± 0.015	0.688 ± 0.086	0.507 ± 0.056
LightGBM	0.737 ± 0.017	0.590 ± 0.024	0.579 ± 0.018	0.666 ± 0.074	0.523 ± 0.061

Note. Values are presented as mean ± SD from 100 repetitions of an 80:20 train-test split. Models were tuned via 5-fold cross-validation (see Supplementary Table S4).

Abbreviations: ROC-AUC, area under the receiver operating characteristic curve; PR-AUC, area under the precision–recall curve; F1, harmonic mean of precision and recall; LR, logistic regression; RF, random forest; XGB, extreme gradient boosting; LGBM, light gradient boosting machine.

Table S7. Comparison of class imbalance handling strategies across four machine learning models.

Method	Model	ROC-AUC	PR-AUC	F1-score	Recall	Precision
Re-weighting	Logistic Regression	0.726	0.565	0.565	0.829	0.429
Re-weighting	Random Forest	0.753	0.603	0.589	0.805	0.464
Re-weighting	XGBoost	0.750	0.606	0.588	0.759	0.480
Re-weighting	**LightGBM**	**0.762**	**0.621**	**0.601**	0.763	0.496
SMOTE	Logistic Regression	0.722	0.564	0.564	0.809	0.432
SMOTE	Random Forest	0.747	0.594	0.586	0.809	0.459
SMOTE	XGBoost	0.748	0.598	0.576	0.844	0.438
SMOTE	LightGBM	0.753	0.612	0.581	0.716	0.489
Undersampling	Logistic Regression	0.727	0.561	0.563	0.782	0.440
Undersampling	Random Forest	0.757	0.593	0.592	0.685	0.521
Undersampling	XGBoost	0.752	0.603	0.599	0.735	0.505
Undersampling	LightGBM	0.757	0.608	0.591	0.669	0.529

Note. All metrics were evaluated on the held-out test set of the primary 80:20 stratified split (random_state = 92). SMOTE and random under sampling were applied to the training set only, while the test set remained unchanged. To ensure a fair comparison, the same hyperparameters selected from the original 5-fold cross-validation were retained for each refit. Bold values indicate the highest value within each column.
Abbreviations: SMOTE, Synthetic Minority Oversampling Technique; ROC-AUC, area under the receiver operating characteristic curve; PR-AUC, area under the precision–recall curve; F1-score, harmonic mean of precision and recall; LR, logistic regression; RF, random forest; XGBoost, extreme gradient boosting; LightGBM, light gradient boosting machine.

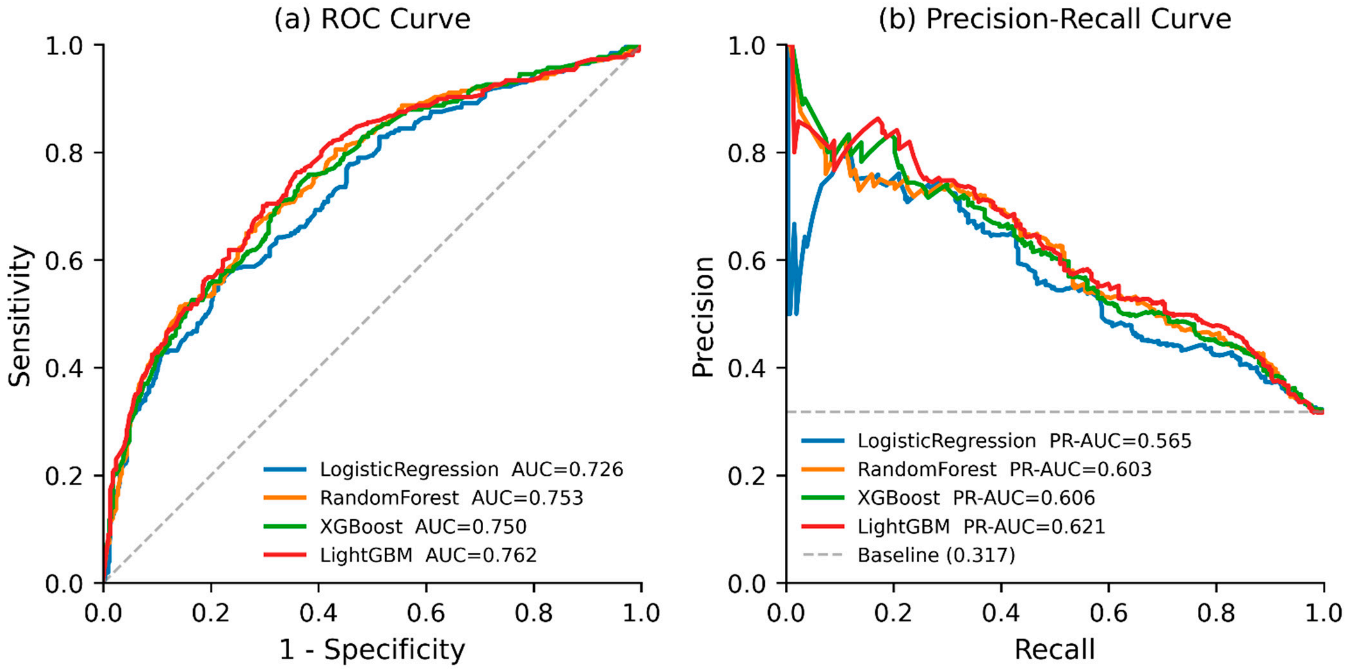

Figure S1. ROC and PR curves of the four machine learning models for CRS prediction.

Note. (a) ROC curves with AUROC values. (b) PR curves with PR-AUC values. Curves were derived from the primary 80:20 stratified train-test split (random_state = 92) on the held-out test set.

Abbreviations: ROC, receiver operating characteristic; PR, precision–recall; AUROC, area under the ROC curve; PR-AUC, area under the PR curve; CRS, cytokine release syndrome; LR, logistic regression; RF, random forest; XGBoost, extreme gradient boosting; LightGBM, light gradient boosting machine.

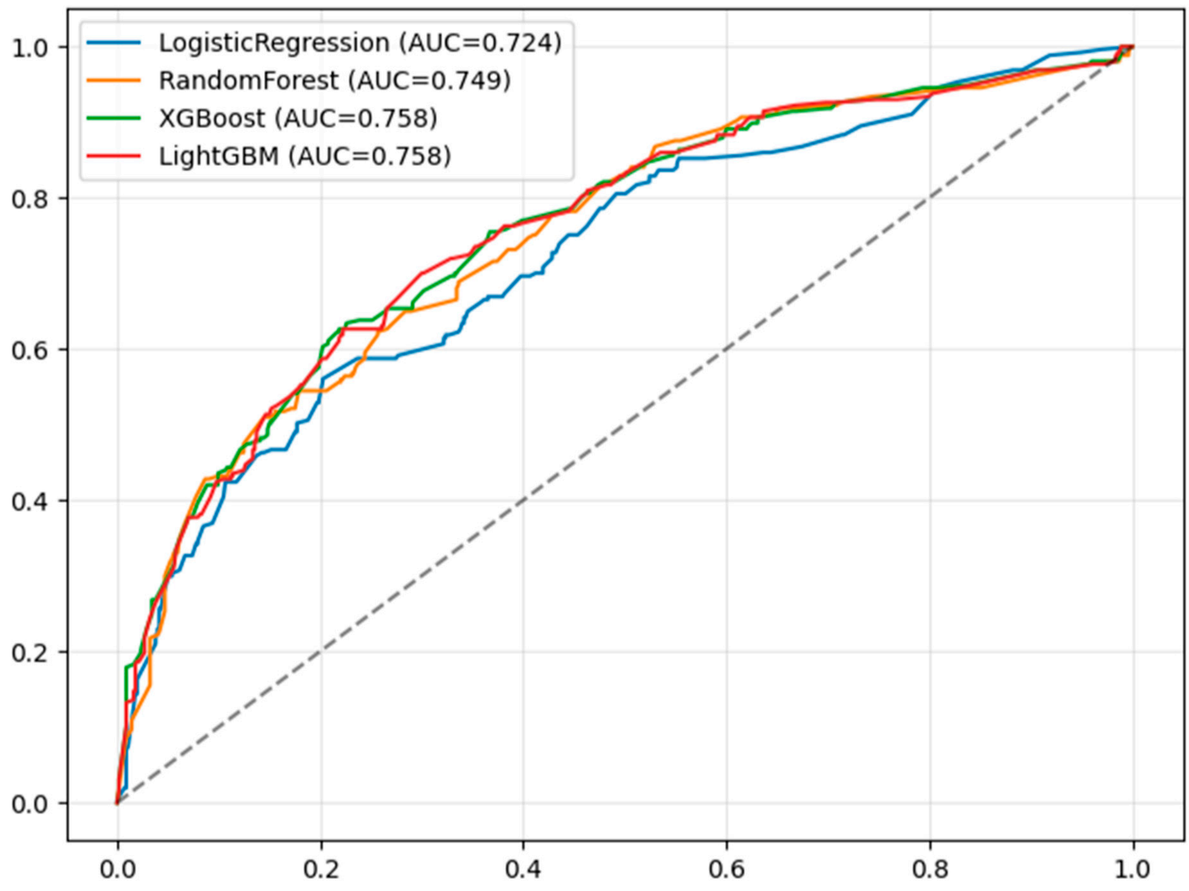

Figure S2. ROC curves of machine learning models for predicting CRS using LASSO-selected features.

Abbreviations: AUC, area under the curve; XGBoost, extreme gradient boosting; LightGBM, light gradient boosting machine; ROC, receiver operating characteristic; CRS, cytokine release syndrome; LASSO, least absolute shrinkage and selection operator.
